# Mechanisms regulating follicle selection in ruminants: lessons learned from multiple ovulation models

**DOI:** 10.21451/1984-3143-AR2018-0027

**Published:** 2018-08-03

**Authors:** Alvaro Garcia-Guerra, Milo C. Wiltbank, Sarah E. Battista, Brian W. Kirkpatrick, Roberto Sartori

**Affiliations:** 1 Department of Animal Sciences, The Ohio State University, Columbus, OH, 43210 USA; 2 Department of Dairy Science, University of Wisconsin-Madison, Madison, WI, 53706, USA; 3 Department of Animal Sciences, University of Wisconsin-Madison, Madison, WI, 53706, USA; 4 Department of Animal Science, University of São Paulo, Piracicaba, SP, 13418-900, Brazil

**Keywords:** follicle selection, high fecundity, ruminants.

## Abstract

Selection of a single dominant follicle from a cohort of growing follicles is a unique biological process, a key step in female reproductive function in monovular species, and lies at the core of reproductive technologies in cattle. Follicle growth and the number of follicles that ovulate are regulated by precise endocrine, paracrine, and autocrine mechanisms. Most of our current understanding about follicle selection focuses on the role of FSH, LH, and the IGF family in follicle growth and selection of the dominant follicle. However, more recently the role of members of the TGF-ß family has been highlighted, particularly in high fecundity genotypes in sheep. Intercellular signaling between the oocyte and granulosa cells (GC) regulates proliferation and differentiation due to actions of bone morphogenetic protein 15 (BMP15) and growth and differentiation factor 9 (GDF9) within the follicle. Mutations that either knockout or reduce the activity of BMP15 or GDF9 have been found to increase ovulation rate in heterozygotes and generally cause severe follicle abnormalities in homozygotes. A mutation in the intracellular kinase domain of the BMPR1B receptor (Booroola fecundity gene) increases ovulation rate in heterozygotes with further increases in ovulation in homozygotes. The physiological mechanisms linking these mutations to increased ovulation rates are still not well defined. A recently identified high fecundity bovine genotype, Trio, causes increased expression of SMAD6, an intracellular inhibitor of the BMP15/GDF9 signalling pathways. This bovine model has provided insights into the mechanisms associated with selection of multiple dominant follicles and multiple ovulations in carriers of fecundity alleles. The present review focuses on the mechanisms involved in follicle selection in ruminants with a special emphasis on the contribution made by multiple ovulation models in both cattle and sheep. The evaluation of multiple ovulation models in ruminants has allowed us to construct a new physiological model that relates changes in the BMP15/GDF9 signalling pathways to the physiological changes that result in selection of multiple dominant follicles. This model is characterized by acquisition of dominance at a smaller follicle size but at a similar time in the follicular wave with multiple follicles acquiring dominance in a hierarchal sequence, delaying FSH suppression and, thus allowing additional follicles to continue to grow and acquire dominance.

## Introduction

Selection of a single dominant follicle from a cohort of growing follicles is a unique biological process and a key step in female reproductive function. Disturbances in this process can lead to anovulation and infertility or, alternatively, multiple ovulations and multiple births. The improvement in our understanding of follicle development and selection has fueled the development of synchronization protocols for fixed time artificial insemination as well as the development of other reproductive biotechnologies such as superovulation and embryo transfer. As a result, these advances have highlighted the importance of optimal follicle growth and selection as a critical step towards achieving reproductive efficiency in livestock species in order to feed a growing world population.

Alterations in follicle selection can lead to the occurrence of codominant follicles and multiple ovulations which are the basis for dizygotic twinning in cattle and sheep. A particularly useful approach, suitable for study of many biological processes, is the examination of abnormal phenotypes. In this regard the occurrence of multiple ovulations in otherwise monovular species provides a physiological model in which the follicle selection mechanism has been altered in such a way to allow multiple follicles to be selected and ovulate. The available multiple ovulation models in sheep (Juengel *et al*., 2013) and, more recently, in cattle (Kamalludin *et al*., 2018) have contributed to the identification of members of the transforming growth factor-ß (TGF-ß) superfamily as key regulators of ovulation rate and prolificacy unveiling a new set of genes, pathways, and autocrine mechanisms with critical roles in the ovarian physiology of ruminants.

The term follicle selection has been widely used in the literature, however there is a lack of consensus on its scope and implications for reproductive function. Selection has been used in relation to terminal growth of antral follicles, however the loss of preantral follicles by atresia during the early stages of folliculogenesis could arguably be included as part of the selection mechanism. Ginther *et al*. (2001) provided a precise definition for the term in relation to monovular species: is the process wherein one follicle develops from a wave of growing follicles and becomes the only follicle with ovulatory capacity. However, this definition does not account for the possibility of multiple follicles being selected. Conversely, the definition of the word selection is: a collection of things chosen from a group of similar things. Therefore, in order to determine whether follicle selection is occurring, two related events need to be observed: 1) a cohort of growing follicles, and 2) the ability of one (or multiple) follicles to continue to grow and acquire the capacity to ovulate while the remaining follicles do not. In the present review we will use the latter definition which provides the opportunity for more than one follicle to be selected, and thus the occurrence of multiple ovulations can be studied in relation to the follicle selection mechanism.

The purpose of this review is to briefly explore the follicular, endocrine and paracrine factors associated with selection of the dominant follicle and more importantly to explore the contribution of multiple ovulation models in ruminants for understanding the follicle selection mechanism. After introducing the genetic models that have been found to increase ovulation rate in ruminants, the review will explore the potential physiology that underlies the increased ovulation rate in both ovine and bovine models.

## Selection of a single dominant follicle

Follicle development is an essential aspect of female reproduction with intricate mechanisms driving all stages of this process. Initial follicle development involves gradual activation of primordial follicles, which occurs throughout the reproductive lifespan of the female. The complex mechanisms involved in primordial follicle activation are slowly becoming clearer with a key role for KIT ligand from the granulosa cells (GC) activating PI3K/Akt/mTOR pathways in the oocyte and eventually blocking the transcriptional machinery that tonically inhibits primordial follicle activation, such as Foxo3a and p27 (Zhang and Liu, 2015; Bertoldo *et al*., 2018; *Kallen et al., 2018* ). Subsequent growth of the primary, secondary, and early antral follicles involves FSH and a series of paracrine factors, such as C-type natriuretic peptide, that stimulate follicle growth allowing the growing follicles to overcome inhibitory pathways, such as the Hippo pathway, ultimately allowing follicles to enter the antral stages of follicle development (*Hsueh et al., 2015* ). The final development stages of antral follicle growth occurs in a wave-like pattern in ruminants, a model first proposed by Rajakoski (1960). The introduction of ultrasonography and the resulting ability to image the bovine ovary allowed for the concept of follicular waves to be revisited and investigated (Pierson and Ginther, 1987; Sirois and Fortune, 1988). Ultrasonography has now provided substantial information on the dynamics of follicle development and has provided convincing data supporting the follicular wave concept. Moreover, the ability to monitor follicle growth patterns has provided the ability to study follicle selection from a dynamic perspective and allow for the evaluation of temporal associations between follicle events and endocrine changes.

A follicular wave is defined as the synchronous growth of a group of small antral follicles, from which a single follicle is selected becoming the dominant follicle, whereas the remainder of the follicles (subordinate follicles) undergo regression (Ginther *et al*., 1989; Adams, 1994). The follicular wave pattern in ruminants is characterized by the development of typically 2 or 3 follicular waves in cattle (Knopf *et al*., 1989), and 3 to 6 waves in sheep (Ginther *et al*., 1995), during each cycle. Traditionally the emergence of the follicular wave has been defined, in cattle, as the day or examination at which the retrospectively identified dominant follicle is first detected at a diameter of 4-5 mm (Ginther *et al*., 1989).

The pivotal event leading to the occurrence of a single ovulation in monovular species, such as cattle, is referred to as follicle selection. The morphological visualization of the follicle selection process has been termed, diameter deviation, and consists of the continuous growth of the future dominant follicle while the subordinate follicles slow their growth rate or completely cease to grow (Ginther *et al*., 1996, 1997a, 2001). In cattle, diameter deviation occurs, on average, between 2 and 3 days after emergence of the follicular wave, and, although there is individual variability, this range appears to be very representative as it has been observed in multiple studies using both *Bos taurus* and *Bos indicus* breeds (Ginther *et al*., 1996; Sartori *et al*., 2001; Gimenes *et al*., 2008). The future dominant follicle is on average 8 to 9 mm at deviation while the largest subordinate follicle is 7 to 8 mm in *Bos taurus* (Ginther *et al*., 1996, 1997a). Conversely, in *Bos indicus* the future dominant follicle and largest subordinate follicle at deviation onset are 5.9 to 7 mm and 5.4 to 5.9 mm, respectively (Sartorelli *et al*., 2005; Gimenes *et al*., 2008; Sartori *et al*., 2016). These data support the idea that the future dominant follicle generally has a size advantage over the largest subordinate follicle. In this regard, a study in *Bos taurus* found that in 76% of 33 waves the future dominant follicle was larger at deviation than the largest subordinate follicle, in 21% they had the same diameter, while in only 3% of the waves the future dominant follicle was smaller (Ginther *et al*., 1997a). Interestingly, despite diameter deviation occurring at similar times after wave emergence in *Bos taurus* and *Bos indicus*, the size of both the future dominant follicle and the largest subordinate are significantly smaller in *Bos indicus*. The precise mechanism that causes *Bos indicus* to have a smaller follicle size at the time of deviation has not been fully elucidated.

Activation of diameter deviation occurs rapidly (<8 h), as shown by the inhibition of follicle growth less than 8 h after FSH suppression and the growth cessation of the largest subordinate follicle within 8 h after the future dominant follicle reaches 8.5 mm in diameter (Ginther *et al*., 1999). As the follicular wave develops in response to the FSH surge, the developing follicles themselves secrete FSH suppressors, mainly inhibin, that causes the circulating FSH to decline as the wave progresses (Gibbons *et al*., 1999b). The role of FSH in the selection process can be summarized through what has been termed the two-way functional coupling hypothesis between the follicles and FSH. This hypothesis states that during the common growth phase all follicles within the wave contribute (i.e. inhibin) to suppress FSH while depending on FSH for continued growth. Once diameter deviation occurs, the future dominant follicle assumes the coupling role by providing the final suppression of FSH, causing it to reach its nadir while acquiring the ability to survive in the face of basal FSH concentrations (Ginther *et al*., 1996, 1997a, 2000b; Ginther, 2000). Evidence for this hypothesis has been provided by the following results:

1) deviation occurs in association with FSH concentration reaching its nadir (Ginther *et al*., 1996, 1997a; Ginther, 2000); 2) initially all the follicles within the wave contribute to the suppression of FSH as seen by the increase in FSH when a portion or all the follicles are ablated (Gibbons *et al*., 1997); 3) depression of FSH concentrations during the common growth phase by treatment with estradiol (E2) inhibits the growth of all follicles in the wave (Ginther *et al*., 2000b); 4) administration of exogenous FSH allows for continuous growth of follicles and rescue of those destined to become subordinate allowing for multiple ovulations (Adams *et al*., 1993); 5) administration of an inhibin antiserum early in the follicular wave results in development of more than one dominant follicle (Kaneko *et al*., 1993; Takedomi *et al*., 1997); 6) removal of the dominant follicle after deviation is followed by an increase in FSH within 1 h and subsequent increase in the diameter of the largest subordinate follicle occurring 3 h after the increase in FSH (Ginther *et al*., 2016); 7) reduction of FSH concentrations at the time of deviation, by either treatment with a steroid-free fraction of follicular fluid or E2, led to a reduction in the growth of the dominant follicle but not the subordinate follicles (Bergfelt *et al*., 2000; Ginther *et al*., 2000b); and 8) treatment with E2 antiserum leads to an increase in circulating FSH and a delay in deviation (Beg *et al*., 2003).

Circulating luteinizing hormone (LH) has also been implicated in follicle selection. Perhaps the most compelling piece of evidence for the role of LH in follicle selection is the lack of continuous growth of the dominant follicle beyond deviation in the absence of LH pulses (Fike *et al*., 1997; Haughian *et al*., 2013). For example, treatment with acyline (GnRH antagonist) during the first follicular wave reduced circulating LH concentrations and prevented diameter deviation and growth of the largest follicle past 9 mm. Interestingly, the reduction of circulating LH did not affect follicle growth up to the onset of diameter deviation (Haughian *et al*., 2013).

Circulating E2 concentrations begin to increase at the time of deviation as a result of increased E2 production by GC of the future dominant follicle (Ginther *et al*., 1997b; Kulick *et al*., 1999). As previously discussed, the increase in E2 contributes to the final suppression of FSH, such that ablation of the dominant follicle at deviation (~8.5 mm) causes an increase in FSH and, if exogenous E2 is administered, the increase in FSH is delayed for a period of time associated with the increase in E2 (Ginther *et al*., 2000a).

Follicle selection and acquisition of dominance not only involves morphological and endocrine changes but also profound changes in follicular fluid, GC and theca cells (TC). Among the intra-follicular components, E2 concentration has been one of the best characterized changes associated with diameter deviation and acquisition of dominance. Intrafollicular E2 has been shown to increase in the future dominant follicle at or immediately after deviation, and this is associated with increased mRNA expression for CYP19A1 (aromatase) in GC (Beg *et al*., 2000; *Luo et al., 2011* ).

Induction of LH receptors (LHCGR) in GC has been proposed as one of the initial signatures of the dominant follicle phenotype (Beg *et al*., 2001; *Luo et al., 2011* ). The induction of LHCGR in GC appears to be stimulated, at least in part, by FSH and is mediated by increases in cAMP (Luo *et al*., 2003; Nogueira *et al*., 2007). First detection of a difference in LHCGR between the future dominant follicle and the largest subordinate was at 8 to 8.4 mm, immediately prior to diameter deviation at ~8.5 mm (Beg *et al*., 2001). More interestingly, it appears that LH pulses are required for induction of LHCGR in GC, as demonstrated by the lack of LHCGR after treatment with acyline 24 h prior to expected deviation (Luo *et al*., 2011). Follicles that have acquired dominance are the only follicles that ovulate after an LH surge, termed ovulatory capacity, and this capacity is preceded by the acquisition of LH receptors in GC (Sartori *et al*., 2001). Acquisition of ovulatory capacity occurs just after follicle selection in *Bos taurus* and *Bos indicus*, even though the size of the selected dominant follicle is quite different (Sartori *et al*., 2001; Gimenes *et al*., 2008; Simõoes *et al*., 2012). Two other factors that are involved in acquisition of follicle dominance are free IGF1, which decreases in the subordinate follicle but remains elevated in the dominant follicle (Beg *et al*., 2000, 2001) due to breakdown of IGF binding proteins (IGFBP) by the IGFBP protease, PAPPA (Rivera and Fortune, 2003) and FGF10 or FGF18 which increase in the subordinate follicles (Gasperin *et al*., 2012; Portela *et al*., 2015).

## Multiple ovulation models in ruminants

### Genetic models of multiple ovulation

The Booroola Merino ewe was the first high prolificacy line described. It originated from a flock of the Commonwealth Scientific and Industrial Research Organization (CSIRO) using triplet, quadruplet, and quintuplet born ewes and a quintuplet born ram obtained from a commercial operation, Booroola, owned by the Seears Brothers in Cooma, NSW, Australia in 1958 (Bindon, 1984). Since then, at least 19 different mutations affecting ovulation rate related to 6 different genes (Table 1) have been described in sheep and more recently a new mutation has been identified in cattle (Kirkpatrick and Morris, 2015).

**Table 1:** High fecundity genotypes in sheep showing affected gene, allele, line and breed, reported ovulation rate and percentage increase over wild type allele in heterozygous and homozygous carriers and proposed functional modifications in the proteins.

			Number of ovulations (% increase relative to controls)		
Gene	Mutated allele	Name/Breed	Heterozygous	Homozygous	Ref.
BMPR1B	FecB^B^	Booroola	2.8 (+85%)	4.6-9.7 (+204% to 439%)	Davis *et al*., 1982; Mulsant *et al*., 2001; Wilson *et al*., 2001; McNatty *et al*., 2017
	-	Mehraban	1.3† (+25%)	NR	Abdoli *et al*., 2013
BMP15	FecX^I^	Inverdale (Rommney)	2.5-3.2 (+35% to 64%)	POF (primary stage)	Braw-Tal *et al*., 1993; Shackell *et al*., 1993; Galloway *et al*., 2000; Davis *et al*., 2001a
	FecX^H^	Hanna (Romney)	2.6-3.2 (+46% to 72%)	POF (primary stage)	Galloway *et al*., 2000; Davis *et al*., 2001a; Hanrahan *et al*., 2004
	FecX^B^	Belclare (Belclare)	3.3 (+70%)	POF	Hanrahan *et al*., 2004
	FecX^G^	Galway (Belclare, Cambridge)	2.7-3.1 (+37 to 42%)	POF	Hanrahan *et al*., 2004; McNatty *et al*., 2004
	FecX^L^	Lac X-mutated (Lacaune)	3.3-7.2 (+69% to +269%)	POF (primary stage)	Bodin *et al*., 2007; Drouilhet *et al*., 2009
	FecX^R^	Raza Aragonesa	2.0 (+46%)	POF	Martinez-Royo *et al*., 2008; Lahoz *et al*., 2011
	FecX^Gr^	Grivette	2.9 (+16%)^*^	4.6 (+81%)	Demars *et al*., 2013
	FecX^O^	Olkuska	2.0 (+32%)	3.3 (+142%)	Demars *et al*., 2013
	FecX^Bar^	Tunisian Barbarine	1.8 (+64%)	POF	Lassoued *et al*., 2017
GDF9	FecG^H^	High Fertility (Belclare, Cambridge)	4.3 (+88%)	POF	Hanrahan *et al*., 2004
	FecG^T^	Thoka (Icelandic)	+32% (lambing rate)	POF (primary/secondary)	Nicol *et al*., 2009; Juengel *et al*., 2013
	FecG^E^	Embrapa (Santa Ines)	1.3 (+10%)^*^	2.2 (+82%)	McNatty *et al*., 2004; Silva *et al*., 2011
	FecG^V^	Vacaria (Ile de France)	2.4-2.5 (+94%)	POF (small antral follicles abnormal)	Souza *et al*., 2014
	FecG^F^	Finnsheep	2.48 (+6%)	2.98 (+28%)	Vage *et al*., 2013; Mullen and Hanrahan, 2014
B4GALNT2^†^	FecL^L^	Lacaune	3.1 (+114%)	4.6 (+214%)	Drouilhet *et al*., 2009
Unknown	FecX^2W^	Woodlands (Coopworth)	2.7 (+25%)	NR - fertile	Davis *et al*., 2001b
Unknown	FecW	Wishart (Romney)	+0.8-1.0 ovulations	NR-fertile	Davis *et al*., 2006
Unknown	FecD	Davisdale (Border Leicester X Romney)	+0.4-0.8 ova	NR - fertile	Juengel *et al*., 2011

* Non-significant increases from wild-type controls. POF = Primary ovarian failure. NR = not reported.

### Ovine models - Role of TGF-β family members

The evaluation of the aforementioned high ovulation rate phenotypes in sheep has led to the identification of specific genes and pathways with previously unknown effects on ovulation rate. Single gene mutations such as Booroola/FecB, Inverdale/FecX^I^, Hannah/FecX^H^ and FecG^H^ led to the discovery of the role of TGF-β signaling in folliculogenesis and ovulation rate. The lines listed above resulted in the identification of two oocyte- secreted factors (OSF), namely bone morphogenetic protein 15 (BMP15), and growth and differentiation factor-9 (GDF9), as key regulators of GC function (Juengel *et al*., 2013). The original Booroola mutation had a non-conservative substitution in the intracellular kinase domain of the BMPR1B receptor (Mulsant *et al*., 2001; Souza *et al*., 2001; Wilson *et al*., 2001), affecting subsequent signaling pathways. Other mutations identified in sheep result in absent, non-functional, or modified forms of BMP15 or GDF9 (Galloway *et al*., 2000; Hanrahan *et al*., 2004).

**Figure 1:**
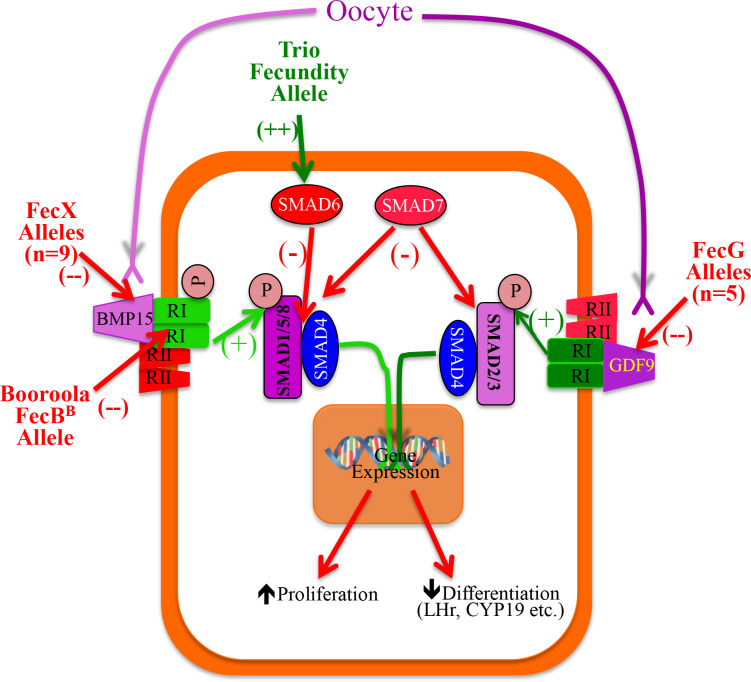
Our current working model of mechanisms in granulosa cells (GC) that lead to reduced follicle growth rate and earlier follicular dominance in carriers of some (n = 16) high fecundity alleles that have been identified in ruminants. The ovine FecX alleles (n = 9) alter the activity or knockout the BMP15 protein; whereas, ovine FecG alleles (n = 5) alter the activity or knockout the GDF9 protein. Both of these proteins are members of the TGF-ß family that are secreted by the oocyte as homodimers or heterodimers and regulate independently or, more likely, cooperatively the proliferation and function of the GC. The ovine Booroola FecB allele causes an alteration in the kinase region of the BMPR1B receptor and thus reduces the activity of the BMP15 pathway. Carriers of the novel bovine Trio allele have a mutation in a regulatory region for SMAD6 causing a dramatic increase in expression of SMAD6 in GC with subsequent inhibition of BMP-15 action by inhibiting Smad-1/5/8 and thus reduces proliferation and allows differentiation.

BMP15 and GDF9 are primarily OSF (Juengel *et al*., 2002), translated as larger precursor proteins, with a pro-region required for proper folding. Upon cleavage by furin-like proteases the mature dimeric form of the protein is produced (McNatty *et al*., 2004; Weiss and Attisano, 2013). Interestingly, GDF9 and BMP15 have been shown *in vitro* to form homodimers and heterodimers, however, unlike most TGF-β members these are not covalently linked by disulfide bonds (Liao *et al*., 2003). As TGF-β members, both BMP15 and GDF9 signal through an assembly of type-I and type-II receptors into heterotetrameric complex receptors (Fig. 1). The type II receptor BMPRII is common to both (Moore *et al*., 2003), however, GDF9 and BMP15 differ in the type I receptor utilized, with GDF9 signaling through ALK5/TGFβRI or ACVR1B/ALK4 (Li *et al*., 2011; Peng *et al*., 2013) and BMP15 signaling through ALK6/BMPRIB (Moore *et al*., 2003). Upon BMP15 binding to its receptor, phosphorylation of the type-I receptor (ALK6/BMPR1B) occurs by means of the kinase domain of the type II receptor, which is constitutively active, ultimately leading to phosphorylation of receptor-regulated SMAD1/5/8 (R- SMAD) and formation of heteromeric complexes of activated R-SMADs with SMAD4 which accumulate in the nucleus and affect gene expression in a cell-type dependent manner (ten Dijke and Hill, 2004; Weiss and Attisano, 2013). Conversely, GDF9 receptor type I (TGFβRI/ALK5) is phosphorylated upon ligand binding and results in the activation of a different set of R- SMADs (SMAD2/3) with subsequent formation of heteromeric complexes with SMAD4 (Gilchrist *et al*., 2006). Two inhibitory SMAD proteins have been described, SMAD6 and SMAD7, which act as negative modulators of BMP15 and GDF9 signalling (Li, 2015). Knockdown of SMAD7 significantly enhanced expression of GDF9-stimulated genes indicating that SMAD7 appears to preferentially inhibit the SMAD2/3 pathway. Conversely, SMAD6 preferentially inhibits BMP signaling by several proposed mechanisms: 1) acting as a SMAD4 decoy thus reducing the formation of SMAD1-SMAD4 heteromers (Hata *et al*., 1998); 2) interaction with type 1 receptors (e.g BMPR1B) preventing phosphorylation of SMAD1/5/8 (*Imamura et al., 1997;*Goto *et al*., 2007); 3) acting as an adaptor protein for Smad ubiquitin regulatory factor 1 (Smurf1) which leads to ubiquitination and degradation of type 1 receptors and R-SMADs (Murakami *et al*., 2003). The high fecundity alleles that act through the GDF9/BMP15 pathways are shown in Fig. 1 (n = 16).

Recently, GDF9 and BMP15 have been shown to act synergistically to regulate GC function, through activation of their receptor complexes as homodimers or heterodimers (Liao *et al*., 2003; McNatty *et al*., 2004). A series of studies have shown that addition of both, recombinant BMP15 (murine, human or ovine) and GDF9 (murine, human or ovine) to murine or rat GC produced a greater effect in GC proliferation than either one alone (Reader *et al*., 2011, 2016; Mottershead *et al*., 2012). Moreover, this effect appears to be mediated by SMAD2/3 activation rather than SAMD1/5/8. Another study also evaluated the effect of recombinant non- purified murine and ovine GDF9 and BMP15 on rat GC (McNatty *et al*., 2005a). Thymidine incorporation indicated that murine GDF9 was the only factor that stimulated proliferation on its own, while ovine GDF9 or BMP15 alone had no effect. However, the combination of murine GDF9 with ovine BMP15 or ovine BMP15 with ovine GDF9 had a dose-dependent synergistic effect on thymidine incorporation, much greater than murine GDF9 alone. In addition, these authors demonstrated that either factor alone had no effect on progesterone (P4) production, but mGDF9+oBMP15 or oGDF9+oBMP15 significantly reduced P4 production in a dose-dependent manner.

The individual and cooperative role of BMP15 and GDF9 has also been investigated in ruminant GC (McNatty *et al*., 2005b). Granulosa cell proliferation was stimulated by oBMP15 and oBMP15+oGDF9 to similar levels in bovine GC, while oGDF9 had no effect and intriguingly mGDF9 inhibited bovine GC proliferation. In ovine GC, addition of the aforementioned factors yielded similar results as those observed in bovine GC; however, the addition of both GDF9 and BMP15 regardless of species of origin resulted in a significantly greater effect on proliferation than with either factor alone. Progesterone production by bovine GC was inhibited by oBMP15 and oGDF9 alone at the highest dose, while combination of both greatly inhibited P4 secretion. In sheep GC, oBMP15 was without effect on P4 secretion, however oGDF9 inhibited while mGDF9 stimulated secretion, and addition of oBMP15 to GDF9 of either species did not modify the effects observed with GDF9 alone (McNatty *et al*., 2005b).

The results previously described emphasize that: 1) both GDF9 and BMP15 regulate GC proliferation and differentiation measured through P4 production *in vitro*; 2) the effect of each factor depends on the species of origin for BMP15 and GDF9, as well as the species of origin of the GC; and 3) a cooperative effect between BMP15 and GDF9 has been observed in all species evaluated, albeit differences appear to exist among species regarding which particular GC function is affected by the cooperation. In addition, recent results indicate that BMP15 and GDF9 proteins can occur as dimers, monomers, and multimers and that components of the immature protein such as the pro-region may have important roles in the biological effects of these OSF (Reader *et al*., 2011; Heath *et al*., 2017). Further, it has been suggested that the dimerization of BMP15 and GDF9 may be occurring at the time of receptor binding with minimal dimerization prior to secretion from GC or within the follicular fluid (Heath *et al*., 2017). It seems clear that further research is needed in this important area in order to establish the precise roles of BMP15 and GDF9 and their dimers in regulation of GC function, particularly with respect to species differences and the possibility that these differences may underlie differences in the number of follicles selected.

### Bovine models

Multiple ovulation models in cattle can be divided into three main types, namely: the USDA- MARC twinner population (Echternkamp *et al*., 2004), the high producing lactating dairy cow (Lopez *et al*., 2005), and the recently identified Trio high fecundity allele (Kirkpatrick and Morris, 2015). Genomic contributions to twinning rate in cattle have been studied by QTL mapping within paternal half-sib families and genome-wide association analyses (GWAS). QTL mapping analyses typically allowed identification of genomic regions potentially associated with twinning rate with broad confidence intervals, making identification of causative genes and polymorphisms extremely challenging. GWAS, by exploiting association across a population rather than within family, potentially narrows localization of underlying genes, though validation is critical. Multiple positional candidate gene regions have been identified by QTL and GWAS analyses (Table 2), though only a few have been replicated.

One of the most replicated regions is the bovine chromosome 5 segment containing IGF1. QTL mapping work in both the Norwegian dairy cattle population and the US Holstein population has provided strong support for a contribution of this genomic region to variation in twinning rate. In a follow-up study, polymorphisms in the IGF1 gene were identified and tested for association in two samples of US Holstein sires representing different timeframes. IGF1 polymorphisms were identified with repeatable associations across these two data sets, one of which was subsequently further validated in the MARC twinner population (Kirkpatrick, 2018: University of Wisconsin-Madison, Madison, WI, USA; unpublished). Two other regions with some degree of replication across studies are the region of bovine chromosome 23 containing the steroid 21-hydroxylase (CYP21) gene and chromosome 7 near the anti- Müllerian hormone (AMH) gene. In all three cases, contribution of these positional candidate genes to genetic variation is uncertain, and further work is needed to establish any causal relationship between these and variation in ovulation or twinning rate.

The search for major genes affecting ovulation in cattle led to the identification of the cow Treble with an exceptional record of prolificacy (Morris *et al*., 2010). This cow produced three sets of triplets in her lifetime among which was a son (Trio) who sired several daughters that had either twin or triplet births, suggesting transmission of a genetic factor across generations. Semen from Trio was used in artificial insemination at the University of Wisconsin-Madison and daughters born to these matings were evaluated for ovulation rate and genotypes for an initial within-family linkage analysis. The linkage analysis provided strong evidence (P < 1x10^-28^) of segregation of a single gene, located on chromosome 10, with a large effect on ovulation rate (1.02 ± 0.08 additional CL per cycle) (Kirkpatrick and Morris, 2015). Examination of this narrowed bovine genomic region suggested multiple candidate genes for the high ovulation rate phenotype. SMAD3 and SMAD6 reside in this region and are part of the TGF-ß signaling system (ten Dijke and Hill, 2004). Considering the mediating roles of SMAD3 and SMAD6 in GDF9 and BMP15 signaling, respectively, and the high fecundity genotypes in sheep associated with GDF9 and BMP15, these two Smads were considered strong candidates for the causative mutation. However, screening for polymorphisms within the coding and flanking regions failed to identify the likely causative polymorphism (Kirkpatrick and Morris, 2015), suggesting that the functional polymorphism was either in a regulatory element located at a greater distance from the gene or is in a different gene in the region. Subsequent gene expression analyses in GC identified and confirmed significant overexpression of SMAD6 in Trio carrier cattle vs non-carrier, strongly implicating SMAD6 as the gene responsible for the high ovulation rate phenotype, though the actual mutation remains unknown (García-Guerra *et al*., 2018a; *Kamalludin et al., 2018* ).

**Table 2:** Chromosomal locations of quantitative trait loci and single nucleotide polymorphisms associated with twinning rate and ovulation rate in cattle.

Trait	Chromosome and approximate location within chromosome (Mb)	Population	Positional candidate genes (chromosome)	Ref.
Ovulation rate	7 (40) and 23 (27)	MARC twinner	CYP21 (23)	Blattman *et al*., 1996
Twinning rate	5 (64), 7 (108), 12 (10) and 23 (26)	Norwegian cattle	IGF1 (5), CYP21 (23)	Lien *et al*., 2000; Meuwissen *et al*., 2002
Ovulation rate	5 (46)	MARC twinner		Kappes *et al*., 2000; Allan *et al*., 2009
Twinning rate	5 (68)	US Holstein	IGF1 (5)	Cruickshank *et al*., 2004; Kim *et al*., 2009b
Twinning rate	8 (108), 10 (26) and 14 (51)	US Holstein		Cobanoglu *et al*., 2005
Ovulation rate	14 (61)	MARC twinner		Gonda *et al*., 2004
Ovulation rate	7 (22), 10 (75) and 19 (42)	MARC twinner	AMH (7), ESR2 (10), IGFBP4(19)	Arias and Kirkpatrick, 2004
Twinning rate	4 (44), 5 (67), 6 (8, 44), 7 (68, 76), 8 (58), 9 (34), 11 (47), 14 (21, 38), 15 (23), 23 (51), and 28 (9)	US Holstein	IGF1 (5)	Kim *et al*., 2009a; Bierman *et al*., 2010
Twinning rate	6 (51), 7 (19), 23 (27)		AMH (7), CYP21 (23)	Weller *et al*., 2008
Twinning rate	24 (40)	Italian Maremmana		Moioli *et al*., 2017

### Physiology underlying multiple ovulation models Ovine high fecundity

Mutations in high fecundity ovine genotypes, in general, result in reduced signaling of the GDF9 and/or BMP15 pathways leading to ovulation of multiple smaller follicles as compared to wild type ewes (McNatty *et al*., 1986b). However, the overall ovarian phenotype depends on the precise mutation and the carrier status of the animal (heterozygous or homozygous). Table 1 shows a comprehensive list of the mutations identified in sheep and the associated ovarian phenotype (ovulation rate, percent increase, etc.) according to their carrier status. Homozygous carriers of high fecundity alleles show two distinct ovarian phenotypes. Approximately 60% of the mutations result in primary ovarian failure (POF) and an infertile phenotype in the homozygous state, due to block of follicle development at the primary or secondary stage. Similarly, immunization of ewes against GDF9 and BMP15 results, in most cases, in an anovulatory state and the presence of normal follicles up to the primary stage but only a few abnormal follicles developing past the primary stage (Juengel *et al*., 2002; McNatty *et al*., 2007). Conversely, immunization with some antigens, resulted initially in increased ovulation rate in some ewes. Taken together, these results indicate that a partial decrease in the availability of BMP15 and GDF9 underlies the occurrence of multiple ovulations.

On the other hand, some of the high fecundity ovine genotypes are fertile when present in the homozygous state. The Booroola/FecB results in a further increase in ovulation rate when present in the homozygous state, with ovulation rates that can reach up to 14, with homozygous ewes being fully fertile (Bindon and Piper, 1986; McNatty *et al*., 2017). This mutation does not result in reduction in BMPR1B mRNA or protein concentrations, but there is diminished receptor activity (Fabre *et al*., 2006). More recently, mutations in BMP15 and GDF9 that do not result in POF and sterility in the homozygous state have been described (Table 1). These mutations have a further increase in ovulation rate when present in the homozygous state, although the increase is not as substantial as the one observed in Booroola (Silva *et al*., 2011; Demars *et al*., 2013). Furthermore, ewes with multiple fecundity mutations have substantial increases in ovulation rate indicating that they are independent and do not cancel each other out or lead to anovulatory phenotypes (Hanrahan *et al*., 2004; Drouilhet *et al*., 2009; McNatty *et al*., 2017).

Among the models proposed to explain the occurrence of multiple ovulations in high fecundity ovine genotypes, one states that multiple ovulations could be the result of increasing the number of follicles available for selection (Baird and Campbell, 1998; Scaramuzzi *et al*., 2011; Monniaux, 2016). Data on antral follicle counts (AFC) from ewes with or without high fecundity genes show considerable variation, with some studies indicating no differences in AFC while others indicate greater AFC in ewes carrying a high fecundity allele. Booroola carrier ewes exhibit similar AFC (≥1 mm) as non-carriers as shown by multiple studies (McNatty *et al*., 1985, 1986b; Henderson *et al*., 1987; Gibbons *et al*., 1999a). In agreement, no correlation between AFC and ovulation rate in the previous cycle was found in Booroola ewes (Driancourt *et al*., 1985). However, a recent study showed lower circulating and intrafollicular AMH levels in Booroola/FecB homozygous ewes than wild type ewes but greater number of follicles≥ 1 mm (Estienne *et al*., 2015). In addition, the authors showed that BMP4- induced AMH production by GC was impaired in Booroola/FecB carriers. The authors postulated a model in which greater number of follicles coupled with reduced AMH production, could increase FSH and LH sensitivity and this would allow follicles to mature at a smaller size and in greater number (Estienne *et al*., 2015).

Follicle populations in BMP15/FecX mutations have been the subject of fewer studies and there appears to be differences between specific lines. Heterozygous ewes for the Inverdale/FecX^I^ mutations, were found to have greater AFC (≥1 and ≥2.5 mm) compared to wild types ewes (Shackell *et al*., 1993; McNatty *et al*., 2009). Conversely, in heterozygous Raza Aragonesa/FecX^R^, another BMP15 mutation, no differences were found in AFC (≥3 mm) or circulating AMH when compared to controls (Lahoz *et al*., 2013, 2014). Reports for other mutations are scarce in the literature, however, reports available on Ile de france/FecG^V^ and Lacaune/FecL^L^ ewes showed no differences in AFC between carrier and wild type ewes (Drouilhet *et al*., 2010; Souza *et al*., 2014). The role of antral follicle numbers on selection of multiple follicles has yet to be elucidated and further research is needed.

Several studies have conclusively demonstrated that ewes carrying high fecundity alleles such as Booroola/Fec^B^ (McNatty *et al*., 1985, 1986b; *Souza et al., 1997* ), Inverdale/FecX^I^ (Shackell *et al*., 1993), Ile de france/FecG^V^ (Souza *et al*., 2014), and Lacaune/FecL^L^ (Drouilhet *et al*., 2010) have preovulatory follicles that are significantly smaller than those observed in non- carrier controls. Ewes carrying high fecundity alleles have fewer GC per follicle, compared to wild type control ewes even when comparing similar-sized follicles (McNatty *et al*., 1985, 1986b, 2017). However, when number of GC in all presumptive preovulatory follicles were taken together, there were no differences in total GC number between wild types, Inverdale/FecX^I^, homozygous or heterozygous Booroola/FecB, or even ewes carrying combinations of these mutations and Woodlands/FecW2^W^ (McNatty *et al*., 1979, 1985, 1986b, 2017). Follicle dynamics have not been evaluated to a great extent between ewes carrying high fecundity alleles and wild type controls with the exception of two studies (Souza *et al*., 1997; Gibbons *et al*., 1999a). Both of these studies found smaller follicle sizes in high fecundity ewes starting 48 h after wave emergence.

In agreement with the smaller preovulatory follicle size, the resulting corpora lutea (CL) of ewes carrying high fecundity alleles (i.e Booroola/FecB or Inverdale FecX^I^) are smaller on an individual basis and have fewer cells than those observed in non-carrier control ewes (McNatty *et al*., 1985, 1986b, 2017; Niswender *et al*., 1990; Shackell *et al*., 1993). However, circulating P4, total luteal weight, luteal cell volume, number of cells/gr of tissue, and average cell dimensions were similar between genotypes (Niswender *et al*., 1990; Souza *et al*., 1997). Overall, high fecundity genotypes ovulate smaller follicles, leading to smaller individual CL, however due to the higher ovulation rate total luteal tissue and circulating P4 is similar.

Two independent studies showed that GC from Booroola/FecB carrier ewes have reduced proliferation compared to wild type ewes (Monniaux *et al*., 2000; Mulsant *et al*., 2001). Furthermore, treatment of GC with BMP4 increased thymidine uptake in wild-types while the same treatment applied to homozygous Booroola/FecB carriers was without effect (*Mulsant et al., 2001;*Fabre *et al*., 2003). Interestingly, the sera of ewes immunized against BMP15 or GDF9 reduced GC proliferation by 70% when sera originated from ewes that were anovular after immunization against BMP15 or GDF9. On the other hand, sera from animals with more than 3 CL inhibited thymidine incorporation by 24-27% (McNatty *et al*., 2007). These results, taken together, provide some evidence that there is impaired signaling of the mutated BMPR1B receptor in Booroola/FecB GC and that in order for multiple ovulations to occur GC proliferation needs to be only partially reduced.

Follicles of ewes carrying the Booroola mutation reached peak aromatase activity and follicular fluid E2 concentrations at progressively smaller diameters related to whether they were heterozygous or homozygous for the mutated allele (McNatty *et al*., 1985, 1986b). Similar to E2, follicles from ewes carrying high fecundity alleles have GC that are responsive to LH stimulation at a smaller follicle size (McNatty *et al*., 1986a, 2009, 2017), are capable of producing levels of cAMP, in response to LH, at smaller sizes (Henderson *et al*., 1987), and have greater mRNA for LHCGR in GC of small (1-3 mm) and medium (3-4.5 mm) follicles than similar follicles from wild type controls (Juengel *et al*., 2017). Thus, acquisition of LHCGR in GC, increased E2 production, and decreased IGFBP (Monniaux *et al*., 2000) at smaller follicle sizes indicate that acquisition of dominance at a smaller follicle size is a hallmark of the physiology of ewes carrying high fecundity alleles.

It is well-known that exogenous FSH can override the selection mechanisms allowing for codominance and multiple ovulations in both sheep and cattle. Initial studies on circulating FSH in Booroola/FecB ewes found elevated mean concentrations in homozygous carriers, while heterozygous carriers were intermediate but not different from controls (McNatty *et al*., 1987; Boulton *et al*., 1995). Similarly, a recent study in ewes containing both the Booroola/FecB and Inverdale FecX^I^, had greater circulating FSH on day 5-6 of the estrous cycle compared to controls (Juengel *et al*., 2017). The differences found in circulating FSH however do not appear to be associated with differences in circulating inhibin (McNatty *et al*., 1992; *Shackell et al., 1993* ; Souza *et al*., 1997). Conversely, other studies found no differences in circulating FSH between Booroola ewes (homozygous and heterozygous combined) and wild type controls (Souza *et al*., 1997; Gibbons *et al*., 1999a). The conflicting results in circulating FSH concentrations between carriers of the Booroola mutation and wild type ewes could potentially result from the use of different breed backgrounds, differences in assays, and finally different strategies to normalize FSH patterns between ewes. In this regard, only two studies evaluated circulating FSH normalized to emergence of the follicular wave and both failed to identify differences in FSH (Souza *et al*., 1997; Gibbons *et al*., 1999a). However, small follicle size and less pronounced diameter deviation in ewes make it reasonable to expect that subtle differences in FSH near follicle selection would go unnoticed.

One study that provided evidence against the role of changes in circulating FSH driving the occurrence of multiple ovulations in ovine, high- fecundity genotypes was done in carriers and non- carriers of Booroola FecB ewes using autotransplanted ovaries, a GnRH antagonist and controlled patterns of FSH and LH in both genotypes (Campbell *et al*., 2003). Interestingly, the differences in ovulation rate between carriers and wild types were maintained despite similar FSH/LH patterns being delivered. The authors stated that these results did not support the idea that the FecB gene acts simply through increasing gonadotropin stimulation but that sensitivity of follicular cells to gonadotropins must also be critical for increased ovulation rate produced by the FecB allele. Thus, descriptive studies on FSH patterns support increases/alterations in circulating FSH in carriers of fecundity genotypes but definitive evidence has not yet been provided, whereas this manipulative study supports the idea that differences in circulating FSH may be secondary to ovarian gonadotropin sensitivity in determining the ovulation rate in carriers and non- carriers of fecundity genotypes.

### USDA MARC twinner cattle

The twinner cattle population at USDA-MARC has been the result of selection over multiple generations for twinning and ovulation rate (Echternkamp *et al*., 1990a; Gregory *et al*., 1990). As a result, twinner cattle have on average 2.1 ovulations per cycle, with ~60% of the cycles having 2 ovulations and rarely exceeding 4 ovulations (Echternkamp *et al*., 2009).

Follicle numbers have been investigated in MARC twinner cattle as a potential component of the mechanism underlying multiple ovulations, as previously described for high fecundity alleles in sheep. Total antral surface follicles are greater in twinner cows than in control cows from an unselected population (Echternkamp *et al*., 1990b, 2004). Furthermore, differences have been found in the preantral follicle population, with twinner cows having significantly more (~2-fold) secondary follicles than controls, while no differences were found in primordial, primary, or tertiary follicles (Cushman *et al*., 2000). However, the control population used for comparison is of paramount importance as follicle numbers have been shown to be highly variable among individuals. The control population utilized in MARC twinner studies may have a different genetic makeup and this could potentially confound reported associations. Twinner cattle preovulatory follicle size (12 h after estrus) was also evaluated in relation to ovulation rate, indicating that individual follicle diameter decreased as ovulation rate increased from 1 to 3, but no further decrease was seen thereafter although sample size of cows with ovulation rates greater than 3 was low (Echternkamp *et al*., 2009). Likewise, individual CL volume decreased with increased ovulation rate, however total CL volume, weight and circulating P4 increased with increasing ovulation rate (Echternkamp *et al*., 2004, 2009). Circulating FSH in the USDA-MARC twinner population did not indicate differences when compared with unselected controls, although interpretation of the results is hampered by lack of normalization to wave emergence or deviation (Echternkamp, 2000; Echternkamp *et al*., 2004). Thus, precise follicle dynamics and associated hormonal patterns of MARC twinner cattle, in relation to diameter deviation, have not yet been conclusively investigated.

Several aspects of the follicular microenvironment have been evaluated in relation to selection of multiple dominant follicles in the MARC twinner cattle population. The IGF1 system appears to be a key component of the altered selection mechanism in MARC twinner cows as evidenced by: 1) greater plasma and intrafollicular IGF1 (Echternkamp *et al*., 1990b, 2004); 2) greater binding activity of IGFBP-3 and one form of IGFBP-5, but lower binding activity of one form of IGBP-4 (Echternkamp *et al*., 2004); and 3) decreased IGF2R mRNA in GC, an IGF2 receptor lacking kinase activity (Echternkamp *et al*., 2012; Aad *et al*., 2013). Thus, lower IGF2R and IGFBP-4 and greater IGF1 have been implicated in selection of additional follicles, although the precise sequence of events has not been resolved.

### Trio, a novel high fecundity allele

The recent discovery of Trio provides an outstanding opportunity to investigate the physiologic mechanisms associated with selection of a single follicle (non-carriers) or multiple follicles (Trio carriers) during different experimental conditions (Kirkpatrick and Morris, 2015). The cow is a particularly useful research model for studies of follicular selection due to ease and accuracy of ultrasound evaluation of bovine follicular growth, ability to evaluate concurrent endocrine profiles, and methods to manipulate or evaluate follicular fluid (Ginther *et al*., 1997b, 2004; Beg *et al*., 2002; Beg and Ginther, 2006).

Evaluation of more than 243 estrous cycles in Trio carrier cattle (heterozygote) and half-sib non- carriers, revealed that Trio carriers had a consistent increase in ovulation rate with a mean ovulation rate of 3.5 ± 0.2 while non-carriers had a mean ovulation rate of 1.1 ± 0.1 (García-Guerra *et al*., 2017b). In Trio carriers, most (70.4%; 95/134) cycles had 3 or 4 ovulations, and few cycles had >5 ovulations (4.4%) or only a single ovulation (5.2%), while ~89% of the cycles of non-carrier controls had single ovulations, and no cycles were observed having 3 ovulations or more. Thus, heterozygous carriers of Trio have a dramatic and consistent increase in ovulation rate. More recently, ovulation rate was obtained from 3 homozygous Trio allele carriers during a minimum of 4 estrous cycles (Garcia-Guerra *et al*., 2018; University of Wisconsin- Madison, Madison, WI, USA; unpublished). Mean ovulation rate per cycle was 4.3 ± 0.5 for homozygous Trio allele carrier heifers, indicating that the Trio allele does not result in primary ovarian failure in the homozygous state, unlike many of the high fecundity genotypes found in sheep. However, due to low number of homozygote animals, it is currently unclear if homozygous Trio carriers have similar or increased ovulations compared to heterozygotes.

We recently investigated differences in antral follicle populations in carriers *vs*. non-carriers of Trio (García-Guerra *et al*., 2017b). Trio carriers had AFC (≥2 mm) at the time of wave emergence that were similar to non-carriers. Furthermore, no association was observed between AFC and mean ovulation rate for the preceding four estrous cycles in Trio carriers or non- carriers despite large variation in AFC. Production of AMH in females is from GC of growing preantral and small antral follicles, thus providing a good representation of the dynamic follicle reserve (*Rico et al., 2011* ). In cattle, AMH and AFC have been found to have relatively high correlations ranging from 0.59 to 0.88 (Ireland *et al*., 2008). As expected, our results indicate that circulating AMH concentrations were positively associated with AFC in both Trio carrier and non-carrier cattle (García-Guerra *et al*., 2017b). However, there was no difference between genotypes in circulating AMH. Thus, differences in size of the antral follicle population is not causing selection of multiple follicles in cattle carrying the high fecundity allele, Trio. Trio carriers exhibit predominantly (>70%) 3 follicular waves during the estrous cycle, similar to non- carriers (García-Guerra *et al*., 2017a). In agreement with increased ovulation rate, each follicular wave contained greater number of dominant follicles in Trio carriers (3 to 4 per wave) compared to non-carriers (1 per wave). In addition, Trio carriers ovulate multiple smaller-sized follicles, however, the study of follicle dynamics in Trio carriers also indicates that follicles are smaller during the entire follicular wave (García-Guerra *et al*., 2017a, 2018b). Evidence for smaller follicle size in Trio carriers arises from the following observations: 1) largest, smallest and mean preovulatory follicle diameter is greatly reduced; 2) future dominant follicles are smaller at wave emergence, at deviation, and at time of maximum diameter; 3) future first subordinate follicle is smaller at wave emergence and deviation; 4) reduced follicle growth rate; and 5) resulting CL are significantly smaller on an individual basis.

Nevertheless, there is clear evidence of subordinate follicles in Trio carriers, supporting the idea that selection still occurs but at a smaller size and with a greater number of selected follicles in Trio carriers. Thus, diameter deviation between future dominant follicles and subordinate follicles occurred at similar times (~3 days) in Trio carriers and non-carriers albeit at significantly smaller follicle sizes (6 mm *vs*. 8.6 mm, respectively).

**Figure 2:**
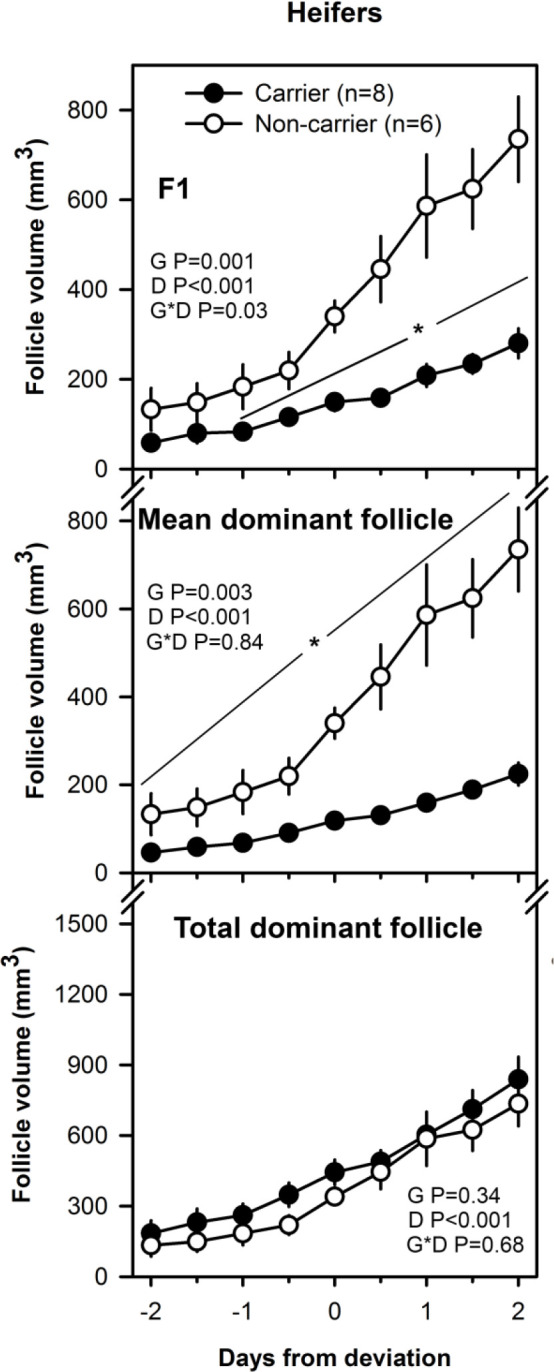
Growth profile of largest follicle volume (F1), mean dominant follicle volume, and total dominant follicle volume in Trio carrier and age-matched, half-sib non-carrier control heifers (adapted from García-Guerra *et al*., 2017b). Data were normalized to deviation and each point represents mean (± SEM). ^*^Indicates significant differences between genotype for a given time point (P < 0.05). G, genotype; D, day; G*D, genotype by day interaction.

Evaluation of CL volume and circulating P4 indicates that despite individual CL being smaller in Trio carriers, total luteal volume and resulting P4 concentrations are not different than those observed in non-carriers (García-Guerra *et al*., 2017b, 2018b). As a result, it is reasonable to evaluate follicle size and growth patterns on a volume basis rather than diameter. As shown in Fig. 2, the diameter and volume of the largest or mean dominant follicle (average of all dominant follicles) normalized to onset of diameter deviation is significantly smaller in Trio carriers compared to non-carrier controls (García-Guerra *et al*., 2018b). However, total dominant follicle volume, calculated as the sum of each individual dominant follicle, is similar between genotypes. Interestingly, there is a close association between ovulation rate and individual follicle size such that Trio carrier cattle have ~4-fold greater number of ovulations than single- ovulating controls but their preovulatory follicles are individually ~4-fold smaller on a volume basis than in controls, thus the total preovulatory follicle volume is similar. In agreement, circulating E2, normalized to day of ovulation, is similar between genotypes (García- Guerra *et al*., 2017a), indicating that combined hormonal output is similar regardless of whether it originates from one large follicle or four smaller follicles.

**Figure 3:**
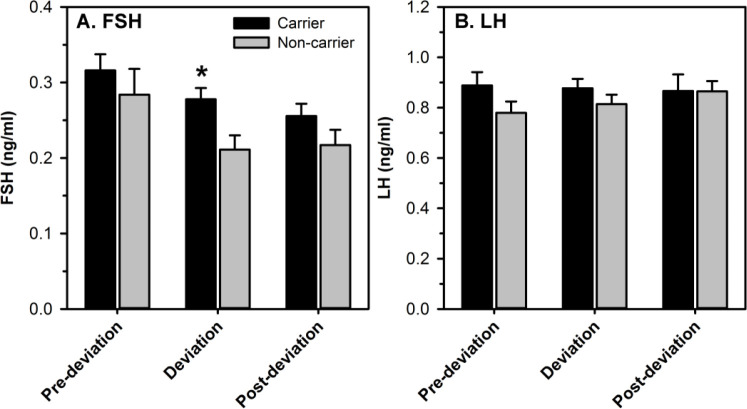
Mean FSH (A) and LH (B) concentrations at 3 distinct time points: pre-deviation (36 ± 12 h before the onset of deviation), at deviation (24 h encompassing the onset of deviation), and post-deviation (36 ± 12 h after the onset of deviation) in cattle with (Trio; n = 13) or without (Non-carriers; n = 9) the high fecundity allele (adapted from García- Guerra *et al*., 2017b). Data represent mean (± SEM). ^*^Indicates significant differences between genotypes (P < 0.05).

The known role of FSH in stimulating follicle growth and the ability to induce multiple ovulations by administration of exogenous FSH (Adams *et al*., 1993), leads naturally to the evaluation of FSH levels in selection of multiple dominant follicles in high fecundity genotypes. In this regard, FSH surges of similar magnitude were found to precede emergence of each follicular wave during the estrous cycle of Trio carriers and non-carriers; although circulating FSH was greater near follicular deviation (García-Guerra *et al*., 2017a). In a second experiment, we synchronized the emergence of the follicular wave and evaluated circulating FSH at frequent intervals *(García-Guerra et al., 2018b* ). Overall, average circulating FSH was greater in Trio carriers than non-carriers. However, precise analysis focusing on the time encompassing observed deviation found that FSH was greater only in a narrow window encompassing deviation (Fig. 3). This elevation in circulating FSH concentrations near the time of follicular deviation is consistent with FSH being a key component of the process that allows selection of multiple dominant follicles in this high fecundity genotype. Nevertheless, provision of large doses of FSH produced a similar superovulatory response in either carriers or non-carriers of the Trio allele (Garcia-Guerra *et al*., 2018; University of Wisconsin-Madison, Madison, WI, USA; unpublished).

Based on the observation that diameter deviation occurs at a similar time but smaller follicle size in Trio carriers, we hypothesized that the dominant phenotype and ovulatory capacity also occurred at a smaller follicle size. We found that follicles of Trio carriers acquired a dominant phenotype, as determined by intrafollicular E2 concentrations, CYP19A1, LHCGR, and PAPPA mRNA abundance in GC, at a significantly smaller size than in non-carrier controls (García-Guerra *et al*., 2018a). As expected, non-carrier single-ovulating cattle acquired a dominant phenotype when follicles were ~8.5 mm. Conversely, Trio carriers acquired a similar phenotype when follicles were ~6 mm, thus supporting the idea that dominance is acquired at a smaller size and in agreement with observed diameter deviation. Moreover, in another experiment, challenge with an exogenous ovulatory stimulus revealed that ovulatory capacity (50% probability of ovulation) was achieved at a reduced follicle size in Trio carriers (5.5 mm) than non-carriers (8.3 mm). Moreover, when each factor pertaining to the dominant phenotype (i.e. E2, LHCGR) were analyzed individually, as a function of follicle size, the 50% probability of dominance was acquired at 5-5.5 mm and 8-8.5 mm for Trio carriers and non-carriers, respectively (García-Guerra *et al*., 2018a). Consideration of follicle size on a volume basis further confirmed our previous findings indicating that acquisition of dominance occurred at a similar time but follicles of Trio carriers were approximately 1/3 to 1/4 the size, on a volume basis, compared to non-carriers.

Finally, based on timing of acquisition of the dominant phenotype, it appears follicles in Trio carriers acquire dominance in a hierarchal manner, as indicated by the increasing number of dominant follicles, based on intrafollicular E2, between days 2 and 4 after wave emergence (García-Guerra *et al*., 2018a). Thus in Trio carriers, the first follicle to acquire dominance would be unable to provide sufficient FSH inhibitor (E2 and/or inhibin), due to its smaller size, to inhibit FSH thus the window for acquiring dominance remains open. As a result, the next follicle(s) in the hierarchy is (are) able to acquire a dominant phenotype and contribute to increasing circulating E2. The metaphorical gate of selection then, would remain open, until a sufficient number of dominant follicles (3 to 4) are present that equate to the same total follicle volume as that observed in a single dominant follicle in non-carrier cattle. This will produce sufficient FSH inhibitor (circulating E2) necessary to provide the final suppression of FSH and thus prevent selection of additional follicles.

## Conclusions - A model for selection of multiple follicles

Several models have been proposed to explain the occurrence of multiple ovulations in high fecundity genotypes in sheep, primarily based on studies of Booroola/FecB ewes (Baird 1987; Scaramuzzi *et al*., 2011; Juengel *et al*., 2013; Monniaux, 2016). Four potential, not mutually-exclusive, mechanisms have been proposed: 1) widening the selection gate by increasing FSH, which translates into a longer period of time during which FSH is above the threshold; 2) increasing the number of follicles, thus increasing the probability that more than one follicle is allowed through the gate; 3) decreasing GC proliferation and size at which follicles acquire LH receptors; and 4) increased sensitivity of follicles to FSH, thus allowing follicles to continue to grow in a lower FSH environment. Our recent data obtained from cattle carrying the high fecundity allele, Trio, are consistent with the first (slight FSH increase) and third (smaller follicles at dominance due to decreased GC proliferation) mechanisms but provide clear evidence against the second mechanism, increased number of follicles, as a mechanism to increase ovulation rate in Trio carriers. Although certain high fecundity ovine genotypes and the MARC-twinner bovine model have been reported to have greater numbers of small follicles, the mere increase in follicle numbers alone does not seem sufficient to explain the occurrence of increased multiple ovulations, since there is still no explanation for why only a portion of available follicles are selected. In addition, substantial variation in follicle numbers has been shown in single ovulating cattle, and no evidence has been provided so far to link this variation with the occurrence of multiple ovulations. Moreover, *Bos indicus* are known to have much greater numbers of antral follicles than *Bos taurus*, however the occurrence of multiple ovulations and births appears to be rare (Sartori and Barros, 2011). In a recent review (Monniaux, 2016), the increase in follicle numbers in regulation of multiple ovulations has been integrated with the fourth proposed mechanism (greater FSH sensitivity). The increase in FSH sensitivity has been hypothesized to arise from changes in number of FSH receptors, decreased AMH, increased response of the receptor upon stimulation, or increase in other factors that modulate or act in conjunction with FSH to stimulate GC (i.e. IGF1). Recent results from Trio carriers did not show an increased expression of FSH receptor or increased intrafollicular IGF1 but found decreased expression of both factors in dominant follicles in Trio carriers as compared to controls (García-Guerra *et al*., 2018a).

The most consistent finding for all high fecundity genotypes is smaller-sized dominant and ovulatory follicles in Trio carriers or ovine fecundity genotypes. In addition, our evidence in Trio, as well as evidence in high fecundity ovine genotypes support another aspect of the third mechanism, related to acquisition of LH receptors in smaller-sized follicles. Thus, development of smaller follicles that acquire dominance and ovulate at smaller sizes appears to be a key component for selection of multiple follicles. Thus, multiple smaller follicles function as a cohesive unit that has the same hormonal output (i.e. E2) and upon ovulation results in multiple CL of smaller individual size but the same total number of luteal cells with corresponding similar P4 output.

An important aspect that stems from the development of smaller follicles in Trio carriers is their reduced growth rate. The reduced follicle growth is likely mediated by a reduction in the rate at which GC proliferate, resulting from attenuation of BMP15 signaling pathways in response elevated SMAD6 (García-Guerra *et al*., 2018a; Kamalludin *et al*., 2018). Thus, overexpression of SMAD6 in GC of Trio carriers would be similar, functionally, to high-fecundity ovine mutations that affect BMP15 and GDF9. However, the precise effects of these factors in bovine GC along with the regulation of such pathways have not been investigated and should be the focus of future research.

**Figure 4:**
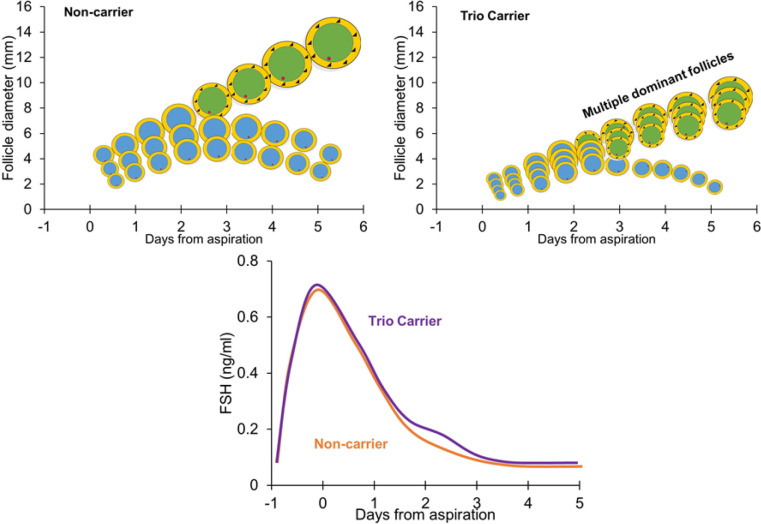
Physiological model for follicle selection in single ovulating cattle (left) and Trio carriers with multiple ovulations (right). The inset below shows the FSH concentrations during the period after follicle aspiration, emphasizing the comparison between carriers and non-carriers of the Trio allele.

The potential for FSH being the major driver for occurrence of multiple ovulations is extremely appealing. Our findings of elevated FSH concentrations, particularly around the time of expected deviation provides clear support for this mechanism, and this would provide a logical physiologic explanation for increased ovulation rate. Thus, our current model (Fig. 4) for Trio carriers is: 1) at wave emergence follicles are smaller (~50% of volume); 2) follicles develop at a reduced rate resulting in follicles ~25-30% of the volume of non-carriers at deviation; 3) upon reaching the decisive period (2.5-3 days) the largest follicle acquires dominance (i.e. LH receptors) with a corresponding increase in E2 production, however because of much smaller follicle size, the resulting E2 production is not sufficient to completely suppress FSH; 4) as a result the next follicle is allowed to acquire dominance (following a hierarchal order) until sufficient dominant follicle volume is reached, resulting in sufficient GC numbers to induce final suppression of FSH. Future studies are needed to definitely evaluate this model in other high fecundity genotypes.
